# From hype to reality: where did the digital health promises go?

**DOI:** 10.1108/IJHCQA-09-2025-0134

**Published:** 2026-04-07

**Authors:** Farooq Mubarak

**Affiliations:** Department of Management and Entrepreneurship, Information Systems Science Unit, Turku School of Economics, University of Turku, Turku, Finland

**Keywords:** Digital health adoption, Digital literacy, Patient safety, Socio-technical framework, Health equity, Participatory design, Low- and middle-income countries, Digital inclusion, Culturally tailored healthcare technology, Health system governance

## Abstract

**Purpose:**

Digital health has been widely heralded as a transformative solution for improving healthcare access and quality, yet evidence from low- and middle-income contexts suggests adoption is uneven and contingent on multiple socio-technical, cultural and infrastructural factors. Karachi presents a critical case to examine these dynamics and their implications for patient safety and equity.

**Design/methodology/approach:**

A mixed methods design was employed, integrating a structured narrative review of literature published between 2020 and 2024 with qualitative interviews conducted among 31 healthcare practitioners in Karachi. The literature synthesis draws on peer-reviewed studies and institutional reports to map global trends, while semi-structured interviews provide context-specific insights into real-world implementation. Interviews explored experiences with digital health tool adoption, patient engagement and perceived barriers to sustainable implementation. Data were analysed using thematic synthesis and thematic analysis, followed by side-by-side integration to identify convergence and divergence between global evidence and practitioner experience.

**Findings:**

The findings reveal that digital health adoption is shaped by interdependent socio-technical constraints rather than purely technological factors. The findings reveal that digital health adoption is shaped by a complex interplay of socio-technical factors rather than technological availability alone. Five interrelated themes define this landscape: technical instability of digital systems, persistent gaps in patient digital skills, cultural and linguistic misalignment, context-dependent trust dynamics and weak policy and system integration. While the pandemic accelerated digital uptake, this expansion was often rapid and uneven, producing short-term familiarity without sustained competence. In certain cases, digital health interventions reproduced existing inequalities, disproportionately benefiting populations with higher digital capacity. Importantly, these limitations are not only barriers to adoption but also sources of clinical risk, as unreliable systems, misinterpretation of information and exclusion from digital pathways can compromise patient safety and continuity of care.

**Research limitations:**

The study is contextually grounded in a single megacity, which may limit direct generalisation, although the patterns identified resonate with broader low-resource settings. Future research would benefit from longitudinal designs to assess whether post-pandemic digital health gains are sustained, and from intersectional analyses that capture how digital exclusion interacts with gender, age and socioeconomic status. There is also a clear need for stronger integration of patient safety metrics within digital health evaluation frameworks.

**Practical implications:**

Effective digital health implementation requires more than technological deployment. Policy frameworks must prioritise digital inclusion through investments in infrastructure, targeted digital literacy interventions, culturally and linguistically adapted platforms and trust-building mechanisms. Health systems must also embed patient safety principles into digital design and governance, ensuring reliability, usability and error prevention across care pathways.

**Social implications:**

The study highlights that digital health is not inherently democratizing. Without deliberate attention to equity, it risks reproducing and amplifying existing social inequalities in healthcare access and outcomes. Digital exclusion functions as a structural determinant of health, influencing not only who can access services but also who can do so safely. Addressing these inequities is therefore central to both public health resilience and social justice, particularly in contexts where healthcare systems are already unevenly distributed.

**Originality/value:**

This study advances digital health scholarship in three ways. It integrates post-pandemic global evidence with practitioner perspectives from a low and middle-income megacity, offering a grounded account of implementation realities often absent from policy-level analyses. It reframes digital health as a socio-technical system rather than a technological intervention, emphasising the centrality of contextual alignment. Most importantly, it introduces patient safety as a core analytical lens, demonstrating that the success of digital health initiatives must be evaluated not only in terms of access or efficiency but also in terms of their capacity to deliver safe, reliable and equitable care. In other words, the study argues that meaningful digital health transformation must balance technical innovation with a commitment to patient and societal well-being.

**Highlights:**

1. Adoption of digital health technologies is shaped by interconnected socio-technical factors.
2. Gaps in digital skills represent a critical bottleneck for safe and effective use.
3. Cultural and linguistic adaptation enhances usability and accessibility.
4. Short-term exposure alone does not ensure sustained engagement.
5. Embedding digital health into policy, workforce and governance frameworks are essential to promote equity in digital health.

## Statement of public interest

Digital health grew rapidly during the COVID-19 pandemic and has often been promoted as a way to address gaps in healthcare access. This study demonstrates that its benefits depend on whether people can use these technologies effectively and trust them. When digital systems are unreliable, hard to navigate, or poorly suited to local contexts, they can delay care and create new risks. Making digital health work for diverse populations requires ongoing investment in skills, infrastructure, and inclusive design. These findings highlight the need to build health systems that are not only technologically advanced but also safe, equitable, and responsive to the needs of real people.

## Introduction

The COVID-19 pandemic marked a historic inflection point for healthcare systems globally, accelerating the adoption of digital health technologies at an unprecedented pace. The integration of digital technologies into healthcare systems has been widely hailed as a transformative shift, potentially improving accessibility, efficiency, and patient empowerment (Lupton, 2014; Sui and Facca, 2020). Over the past decade, the concept of digital health which encompasses telemedicine, mobile health (mHealth) applications, wearable devices, and artificial intelligence (AI)-driven diagnostics has evolved from a peripheral innovation to a central policy priority in many countries. While this growth reflects widespread optimism, the effects of digital health are uneven, potentially empowering some users but creating new challenges and risks for others (Lupton, 2017). The dual reality of digital health, encompassing both its promise and persistent challenges, became especially visible during the COVID-19 pandemic, which exposed limitations in conventional healthcare delivery models and created an urgent demand for remote, technology-enabled solutions (Yang *et al*., 2022). However, despite the global momentum, digital health must also be understood in relation to patient safety, a core dimension of quality care that determines whether digital interventions ultimately improve or compromise clinical outcomes. The World Health Organization (World Health Assembly, 2019) underscores that safe care depends on multiple system-level factors, including reliable information flows and effective communication, increasingly mediated by digital health infrastructure and shaped by health literacy.

Despite decades of investment, the pandemic revealed both the transformative potential and the persistent limitations of digital health systems, particularly in low- and middle-income countries (LMICs). While the potential of digital health to reduce physical, temporal, and financial barriers is widely recognized, evidence suggests that its promise is unevenly realized, particularly in LMICs, where structural inequities, limited infrastructure, and variable digital literacy constrain equitable adoption (Kickbusch, 2001; Norman and Skinner, 2006). This tension between technological promise and real-world implementation constitutes a critical research gap. Importantly, this gap is not only about technological underperformance but also about the misalignment between rapid digital expansion and the socio-technical readiness of populations and health systems in diverse contexts. Crucially, each of these readiness challenges (literacy, infrastructure, interoperability, and trust) has direct implications for patient safety, as inadequate digital capacity risks delays, misinformation, diagnostic inaccuracies, and inequitable access to timely care (World Health Assembly, 2019). Empirical evidence supports this: health-worker uptake of digital health tools in LMICs remains low when facilitating conditions are insufficient (lack of infrastructure, support or training) (Wang *et al.*, 2024). Moreover, the adoption of digital health technologies has been shown to exacerbate inequities, with disadvantaged populations being less able to access or benefit from digital health services (Yao *et al.*, 2022).

Historically, optimism surrounding digital health has been grounded in the belief that technology could level the playing field by reducing disparities in access to healthcare resources (van de Vijver *et al.*, 2023; Gann and Grant, 2013). During the pandemic, this premise appeared to gain traction: governments and private actors rapidly deployed mobile applications for contact tracing, teleconsultations, and vaccination verification, while digital health literacy received unprecedented attention in both academic and policy arenas (Sentell *et al.*, 2020). Substantial financial investments followed, with the global digital health market valued at over USD 200 billion in 2022 and projected to exceed USD 600 billion by 2030 (Grand View Research, 2025). The rapid scale-up of these tools also highlighted how insufficient attention to safety protocols (such as system reliability, error reporting mechanisms, data accuracy, and culturally appropriate interface design) can generate new forms of clinical risk.

Yet, beneath this surge in activity lies a more sobering reality. Many pandemic-driven digital health initiatives failed to achieve their stated objectives, particularly when implemented rapidly without sufficient attention to local contexts. Recent studies indicate that the benefits of digital health have often been unevenly distributed, favoring individuals with higher socioeconomic status, greater digital literacy, and better access to infrastructure (Aissaoui, 2022; Ahmed *et al*., 2023). In LMICs, where structural inequities in healthcare are compounded by infrastructural and linguistic barriers, digital health adoption remains fragmented and frequently exclusionary (Zakar *et al*., 2021). Such inequalities do not only restrict access; they also elevate the risk of unsafe care when vulnerable populations are unable to navigate digital interfaces, misinterpret clinical information, or are excluded from digitalized service pathways that replace traditional options.

This gap between the promise of digital health and its real-world outcomes raises several critical questions. First, to what extent did pandemic-driven digital health innovations meaningfully address pre-existing health inequities? Second, how have structural determinants (such as broadband access, device ownership, cultural norms, and language) shaped the effectiveness of these interventions? Third, what lessons can be drawn to ensure the sustainable integration of digital health into healthcare systems? An additional question that emerges from a patient safety perspective is how digital expansion can be aligned with the principles of safe, effective, and person-centered care; for example, principles that require the minimization of harm and the establishment of robust mechanisms for clinical reliability (World Health Organization, 2021).

Before the pandemic, digital health adoption followed a gradual trajectory, often constrained by infrastructural gaps, regulatory limitations, and uneven digital literacy among patients and providers. COVID-19 disrupted this gradual evolution, forcing healthcare systems to implement telemedicine, mobile vaccination records, AI-assisted diagnostics, and wearable health monitoring devices within months rather than years. This rapid transformation highlighted the adaptability of healthcare systems under pressure but also exposed structural vulnerabilities, socio-technical gaps, and inequities that technology alone could not resolve. These vulnerabilities amplified patient safety concerns, as clinicians and patients were pushed into digitally mediated interactions without adequate training, quality assurance frameworks, or safeguards to prevent errors.

Despite the promise of digital health as a democratizing force, evidence suggests that technology adoption often fails to translate into equitable health outcomes. Both the literature and practitioner experiences indicate that several factors are crucial for effective uptake. These include digital literacy, cultural and linguistic relevance, trust in digital systems, and supportive policy frameworks. In other words, digital health technologies do not automatically reduce health disparities; their success depends on the inclusiveness, integrity, and readiness of the socio-technical ecosystem into which they are introduced. This underscores that digital health must be understood not only as a technological intervention but as a complex socio-technical system whose success hinges on contextual alignment. From a safety standpoint, this means that digital tools must be integrated with attention to human factors, workflow coherence, and error prevention; elements that determine whether digital interventions ultimately enhance or undermine safe clinical practice.

### Global context and case comparisons

The challenges of transitioning to digital health are not limited to resource-constrained settings. For example, in the United Kingdom, the rapid deployment of the NHS COVID-19 app encountered issues related to data privacy, usability, and public trust, which ultimately hindered its uptake (Polzer and Goncharenko, 2022). Similarly, in the United States, telehealth usage surged during the pandemic but has since plateaued, with persistent gaps in adoption among older adults, rural populations, and racial minorities (Walker *et al*., 2020). India's Ayushman Bharat Digital Mission (ABDM) offers a contrasting case, aiming to build a comprehensive national digital health ecosystem; however, even this ambitious initiative contends with digital illiteracy and uneven internet penetration (Ministry of Health and Family Welfare, 2023). Across these contexts, policymakers increasingly recognize that digital health systems require explicit safety standards (for instance, data accuracy requirements, clinical validation processes, and equitable access safeguards) to prevent unintended harms and ensure reliability during clinical decision-making.

These examples underscore that the transition to digital health is not merely a technical challenge but a socio-technical one. Its success depends on the alignment of technological capabilities with human factors such as trust, cultural relevance, and digital literacy. As Kickbusch (2001) argued, health literacy is not simply a matter of information availability but of enabling people to navigate, interpret, and act upon health information meaningfully. In the digital era, this principle becomes even more crucial: digital health literacy requires competencies in both health and technology, rendering it particularly vulnerable to existing inequities. Consequently, patient safety outcomes become tightly coupled with digital health literacy; individuals who cannot interpret digital information or operate digital tools correctly face a greater risk of misinformed decisions, delayed treatment, or inappropriate self-management.

Taken together, these global cases demonstrate that even well-resourced systems grapple with adoption challenges, which reinforces the urgency of examining how these dynamics unfold in LMIC contexts where structural limitations are more pronounced. While these international cases highlight common challenges, examining Karachi for this study (refer to method section for details) allows for understanding how these dynamics manifest in LMIC settings, where infrastructural and socio-cultural constraints are often more pronounced. These contextual constraints also heighten patient safety vulnerabilities, making it critical to explore how practitioners in LMIC megacities negotiate safety risks within digitally mediated care environments.

## The persistence of the digital divide

Despite increased global internet penetration (which reached 67% of the world's population in 2023 (International Telecommunication Union, 2023)), the digital divide remains a structural barrier to equitable healthcare access. This divide is multidimensional, encompassing disparities not only in hardware and connectivity but also in skills, confidence, and alignment of digital services with cultural norms (Aissaoui, 2022). Crucially, even in contexts of widespread access, inequalities in use can persist; studies show that simply distributing devices does not guarantee equal engagement (Walker *et al*., 2020).

Language barriers further complicate digital health adoption. The predominance of English-language health content disadvantages non-English speakers, especially in regions with lower literacy rates (Kickbusch, 2001). During COVID-19, the rapid proliferation of mobile health applications often occurred without adequate localization, leaving substantial portions of the population unable to use these tools effectively (Zakar *et al*., 2021).

Thus, the digital divide is not solely a matter of connection or device availability; it is deeply rooted in socio-cultural, linguistic, and economic inequalities that shape actual usage and benefit realization. Given these disparities, digital exclusion becomes an indirect determinant of patient safety: those who cannot access or effectively use digital tools may experience delays in accessing care, misunderstanding of clinical instructions, or inability to navigate essential health services.

### Local context and study gap

While the global discourse on digital health has expanded, empirical investigations often remain concentrated in high-income countries, leaving a gap in understanding how these technologies perform in LMIC settings. Specifically, despite these global insights, there remains a lack of empirical evidence on how digital health adoption unfolds in LMIC megacities particularly regarding socio-technical determinants and practitioner perspectives. Furthermore, few studies explicitly examine how these socio-technical determinants influence patient safety in digitally mediated care pathways, despite safety being an essential dimension of effective health system performance (World Health Organization, 2021; Wilson *et al.*, 2024).

This study addresses this gap by focusing on Karachi, Pakistan, a megacity that exemplifies many challenges faced by LMICs: high population density, infrastructural constraints, diverse linguistic and cultural groups, and uneven access to digital resources. Karachi serves as a microcosm for understanding broader global digital health dynamics, allowing insights from a localized context to inform wider debates about equity, adoption, and implementation. Moreover, while the body of research on digital health is substantial, significant gaps in knowledge continue to exist:

1. Limited integration of post-pandemic empirical data with practitioner perspectives in LMIC contexts.
2. Insufficient focus on socio-technical determinants, including digital literacy, cultural relevance, and trust.
3. Lack of actionable recommendations linking technology adoption to equitable health outcomes.

Moreover, existing scholarship often frames LMIC challenges as technological deficiencies rather than examining the deeper socio-technical systems that mediate uptake. This study responds to this gap by explicitly situating digital health adoption within the interplay of structural conditions, cultural norms, and practitioner experiences. In addition, this study recognizes that safe and effective care cannot be achieved without understanding how digital barriers (in particular, misinformation, usability problems, and systemic fragmentation) create risks of clinical error and patient harm.

The study makes two key contributions. First, it provides a systematic analysis of the real-world impacts of pandemic-driven digital health adoption, capturing both successes and limitations. Second, it centers digital health literacy and equity as essential considerations in health system transformation. The central argument advanced here is that digital health; while inevitable in its long-term trajectory, cannot fulfill its promise without first addressing the socio-technical prerequisites for equitable adoption. These include robust infrastructure, culturally and linguistically adapted platforms, digital skills training, and sustained public trust-building. Without these foundations, digital health risks reinforcing, rather than dismantling, existing inequities. Therefore, technological innovation alone is insufficient; it must be embedded in inclusive, context-responsive ecosystem strengthening. By integrating patient safety as a cross-cutting concern, this study contributes a critically underexplored dimension: how digital health initiatives can support or compromise safe care delivery depending on their design, implementation, and contextual fit.

**Figure 1:**
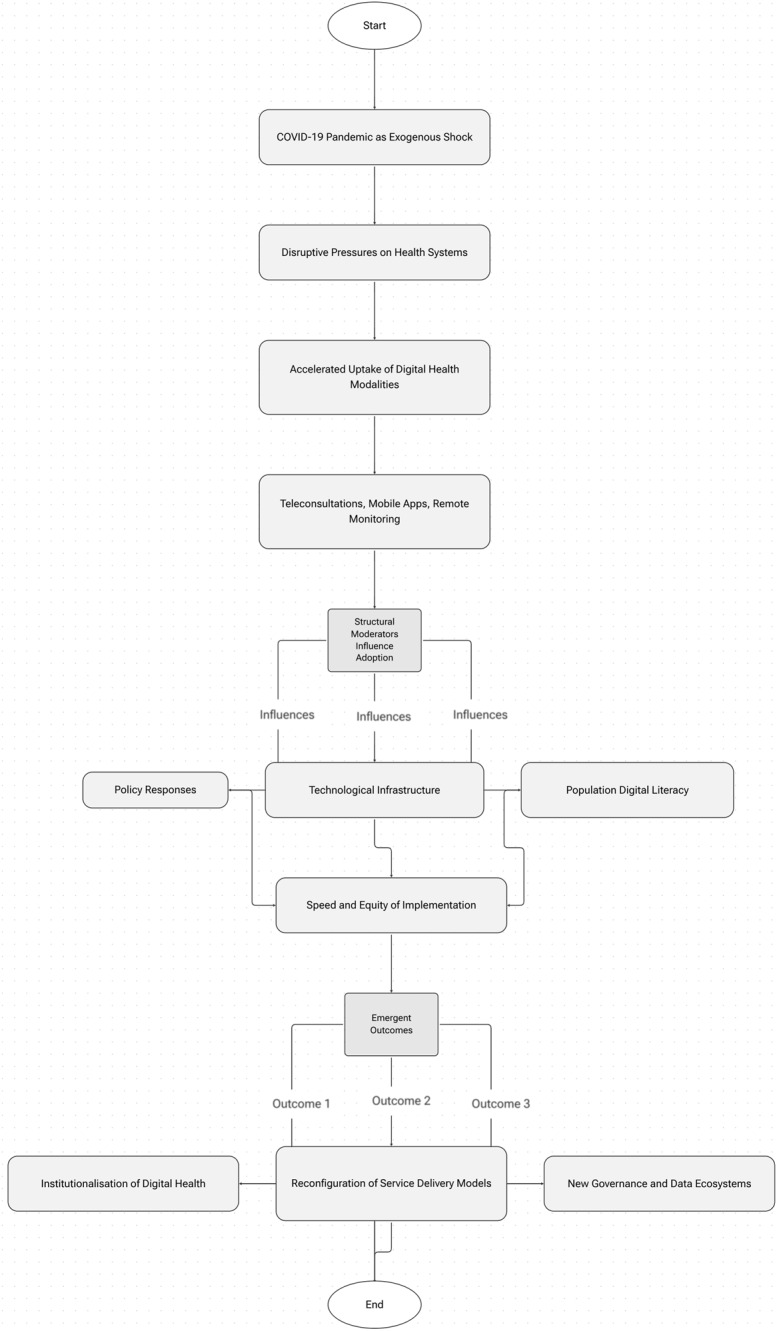
Conceptual model of rapid digital health transformation

To frame this analysis, Figure 1 presents a conceptual model illustrating how the COVID-19 pandemic acted as an exogenous shock that catalyzed rapid digital health transformation. The model outlines a pathway beginning with the pandemic's disruptive pressures on health systems, which triggered accelerated uptake of digital health modalities such as teleconsultations, mobile applications, and remote monitoring. These adoption processes were simultaneously shaped by structural moderators (including policy responses, technological infrastructure, and population digital literacy) that influenced the speed and equity of implementation. The final stage highlights the emergent outcomes: the institutionalization of digital health within routine care, the reconfiguration of service delivery models, and the emergence of new governance and data ecosystems. This framework provides a theoretical anchor for the review and clarifies the mechanisms through which digital health evolved during the pandemic. However, the model also implicitly underscores safety considerations; particularly how system reliability, data integrity, and the capacity of users to engage effectively with digital platforms mediate the overall safety of digitally enabled care.

### Purpose and contribution of this study

This study aims to bridge the gap between global digital health discourse and local practice by integrating empirical insights from healthcare practitioners in Karachi, Pakistan, with a synthesis of post-pandemic literature. In this context, the study highlights how local structural, cultural, and linguistic factors interact with broader global trends, illustrating the socio-technical challenges of digital health adoption in LMICs. Specifically, the study seeks to:

1. Identify the barriers and facilitators to digital health adoption in an LMIC context.
2. Examine how socio-technical factors (digital literacy, trust, cultural fit, and policy alignment) influence technology uptake.
3. Provide evidence-based recommendations for policy, practice, and research to enhance equitable adoption of digital health technologies.

Based on experts' guidance during peer-review process and in light of the recommendation, this study also introduces an additional objective: to explore how digital health adoption interacts with patient safety principles and the delivery of safe, effective care in a low-resource urban context, thereby expanding the analytical lens through which digital transformation is assessed.

Combining global evidence with local insights, this study offers a novel, integrative perspective on digital health adoption, highlighting both the promise of technology and the persistent inequities that may limit its impact. The findings are relevant not only for Pakistan but also for other LMICs grappling with similar challenges, offering a roadmap for designing inclusive, culturally sensitive, and sustainable digital health systems. In doing so, the study provides a clearly delimited and contextually grounded contribution: it examines digital health adoption through the lens of socio-technical equity in a single LMIC megacity, using practitioner insights to illuminate gaps that global, policy-level analyses often overlook. Explicitly incorporating patient safety into this lens, the study extends current scholarship by demonstrating why safe care must be considered a foundational outcome of digital health initiatives rather than an assumed by-product of technological innovation.

## Methods

### Study design

This study employed a convergent mixed-methods design (Creswell and Plano Clark, 2018) integrating a structured narrative literature review with qualitative interviews among healthcare practitioners in Karachi, Pakistan. The structured narrative review synthesizes recent post-pandemic literature (2020–2024) while incorporating seminal pre-2020 studies to provide historical and conceptual context. *Specifically, the literature was considered published between 1990 and November 2025*. The qualitative interviews provide empirical insights from practitioners on the ground, enabling a nuanced understanding of local barriers that reflect global patterns in low- and middle-income countries (LMICs). The convergent design was chosen because it enables simultaneous examination of global evidence and local practitioner perspectives, thereby strengthening analytical triangulation and ensuring that themes emerging from the literature could be directly compared with primary qualitative data.

In line with best practices for mixed-methods health research, the use of a convergent design was also intended to enhance interpretive depth by allowing qualitative findings to contextualize, challenge, or extend evidence derived from the literature. This approach is increasingly recommended in digital health scholarship, where socio-technical systems are complex and cannot be adequately captured by a single form of evidence. By integrating both data strands during interpretation rather than sequentially, the design supported a more holistic understanding of how digital health is adopted and experienced in LMIC contexts, particularly in rapidly evolving post-pandemic environments. Furthermore, this design choice reflects the methodological guidance of contemporary implementation science frameworks, which emphasize the need for combining global knowledge with local experiential insight when evaluating digital transformations in health systems.

Additionally, the convergent approach ensured that practitioner narratives were not treated merely as supplementary data but as an analytically equal component; an important consideration given the study's aim to foreground on-the-ground experiences that often remain underrepresented in global digital health policy discourses. This alignment between empirical voices and published evidence strengthened the study's internal validity and relevance to diverse LMIC settings seeking to interpret global digital health trends through contextually grounded perspectives.

### Rationale for context selection

Karachi was selected as the empirical setting due to its dense population, socio-economic diversity, and representation of typical urban LMIC healthcare challenges. While findings are localized, they capture barriers such as digital literacy gaps, infrastructural limitations, cultural and linguistic mismatches, and trust concerns, which are consistent with global patterns in digital health adoption. Karachi's multilingual environment, uneven distribution of healthcare facilities, and variability in digital readiness across provider types further position it as a strategic site for examining how global digital health ambitions translate into practice. This enables the study to provide lessons relevant beyond Karachi while maintaining empirical rigor. Moreover, as a megacity with both advanced tertiary hospitals and under-resourced primary care clinics, Karachi reflects the complexities and contradictions that characterize digital health implementation in many LMICs.

As further justification, it is important to note that Karachi also offers a concentrated microcosm of socio-technical inequalities that are rarely observable at such scale in a single metropolitan setting. The coexistence of technologically advanced private hospitals with resource-limited public facilities allows for examination of how digital health solutions perform across contrasting infrastructural baselines. Furthermore, Karachi's rapid post-pandemic digitalization initiatives (for example, telemedicine pilots, e-pharmacy expansion, and mobile health awareness campaigns) provide a timely backdrop for investigating adoption dynamics.

In brief, Karachi's stratified health ecosystem offers an ideal empirical site for examining how digital health intersects with structural inequity, governance weaknesses, and socio-cultural norms. By situating the study in a megacity marked by both innovation and infrastructural fragility, the research is positioned to unpack how digital health tools are shaped not only by technology but also by broader political-economic forces. This contextual specificity provides analytical depth while supporting theoretical generalization to other LMIC contexts undergoing uneven digital transformation.

### Literature search strategy

The literature review followed a structured search strategy. Google Scholar, PubMed, World Health Organization (WHO), and Organization for Economic Cooperation and Development (OECD) reports were consulted. Key search terms included digital health, digital literacy, COVID-19, telemedicine, and low and middle income countries (LMICs). Inclusion criteria comprised studies published between 2020 and 2024, reflecting a post-pandemic focus, with seminal pre-2020 works included for contextual grounding; peer-reviewed empirical studies, reviews, and grey literature from global organizations; and content addressing barriers, adoption, or equity in digital health. Exclusion criteria included non-English publications, studies lacking relevance to health system adoption, and non-peer-reviewed opinion pieces.

Google Scholar generates a high volume of heterogeneous, duplicate, and often irrelevant results; an issue recognized in methodological analyses (e.g. Haddaway *et al.*, 2015). Therefore, an additional screening protocol was implemented to ensure rigor and relevance. Search results were first sorted by relevance and publication date. For each search string, the first 200 results were screened by title and abstract, and items that did not explicitly address digital health adoption, literacy, equity, or implementation challenges were excluded. Duplicate entries, which are common in Google Scholar, were manually identified and removed. Grey literature from reputable global health organizations was included only when it directly contributed conceptual or contextual value. This process ensured that the breadth of Google Scholar's coverage was utilized without compromising the specificity, quality, or methodological consistency of the review.

Search results were screened in two stages: first by title and abstract, followed by full-text assessment. The structured narrative approach allowed synthesis of diverse evidence while maintaining a clear conceptual line from pre-pandemic foundational work to post-pandemic empirical insights. In particular, the search strategy adhered to recognized standards for narrative reviews by ensuring transparency in database coverage, keyword construction, and screening principles. Although not a systematic review, efforts were made to enhance reproducibility by documenting search strings, date ranges, and inclusion logic. To further strengthen methodological robustness, backward and forward citation tracking was conducted for highly relevant studies, enabling identification of influential works not captured in database queries. Grey literature inclusion was guided by explicit evaluative criteria; credibility of the issuing organization, methodological clarity, and relevance to LMIC digital health contexts, thereby reducing the risk of incorporating low-quality evidence. These supplementary steps were incorporated to address concerns regarding comprehensiveness and to align the review with best practices for narrative synthesis in global health research.

It is important to clarify that the heterogeneity of global digital health literature (spanning policy documents, empirical studies, conceptual papers, and multi-country assessments) necessitates a flexible synthesis method capable of integrating diverse evidence forms. A systematic review would risk excluding influential grey literature and policy-focused insights that shape digital health implementation in LMICs. The narrative approach thus allowed for a more holistic and contextually grounded synthesis, responsive to the dynamic and rapidly evolving nature of digital health research during and after the COVID-19 pandemic.

### Qualitative interviews

Purposive sampling ensured participants were currently engaged in clinical practice and had direct experience with, or exposure to, digital health applications during and after the COVID-19 pandemic, thereby capturing diverse, practice-based perspectives on adoption and use. The final sample consisted of 31 participants: 21 male and 10 female healthcare professionals, including general practitioners, specialists, and allied health staff. Ages ranged from 28 to 56 years, with an average of 14 years in practice. Recruitment occurred through professional networks, snowball sampling, and outreach via medical associations. This sampling strategy facilitated inclusion of practitioners across different tiers of the health system, ensuring representation from both public and private sectors. This diverse sample supports the convergent mixed-methods design by providing local empirical insights that complement the literature review.

Semi-structured interviews were conducted between October 2022 and April 2023. Due to participant preference and pandemic-related restrictions, most interviews took place face-to-face in clinic offices; a minority were conducted via secure video conferencing platforms. Each interview lasted between 45 and 75 min. A semi-structured guide was used, with open-ended questions probing experiences with digital health tools during COVID-19, perceived benefits and shortcomings of these tools, barriers to patient adoption, and recommendations for improving digital health in the local context. The guide remained flexible, allowing interviewers to pursue emergent lines of inquiry while ensuring core topics were consistently explored across participants. The interviews were conducted in English or Urdu, depending on participant comfort. Urdu responses were translated into English during transcription. All participants provided informed consent and were assured of confidentiality. Transcription was carried out verbatim, and translated segments were checked for accuracy and contextual fidelity to minimize loss of meaning.

It is worth noting that saturation was reached by the 27th interview, as no new substantive themes emerged thereafter. The remaining interviews contributed additional nuance and strengthened confirmatory validity. This aligns with established qualitative guidance suggesting that conceptually rich themes in homogeneous professional groups typically stabilize within 20–30 interviews. Moreover, purposive heterogeneity (especially across clinical specialties and health system tiers) ensured that the sample captured the structural and experiential diversity necessary to answer the research questions (RQs). Additional detail was also incorporated into interviewer training procedures: interviewers underwent a calibration session to standardize probing techniques and ensure consistency in data elicitation across the research team, further enhancing methodological rigor.

### Data analysis

The literature review was thematically synthesized to identify cross-cutting patterns and emergent barriers. Specifically, the corpus was analyzed using a thematic synthesis approach (Thomas and Harden, 2008), enabling the integration of heterogeneous evidence into cross-cutting themes, including infrastructural constraints, trust and governance issues, and gaps in digital skills. Interview transcripts were imported into NVivo 12 for coding and analyzed using thematic analysis, with intercoder reliability confirmed (Cohen's κ = 0.84). Coding followed an iterative process: initial inductive codes were generated from the data, then refined through team discussion to form higher-order themes. Discrepancies were resolved through consensus to ensure analytical consistency and transparency.

To integrate the datasets, a “side-by-side” approach was employed: literature-derived themes and interview findings were compared, highlighting areas of convergence, divergence, and context-specific patterns. Each theme was explicitly linked to the research questions: (1) What are the barriers to equitable digital health adoption? (2) How do infrastructure, skills, trust, and cultural factors influence adoption? (3) What strategies could enable sustainable integration of digital health solutions? This integrative comparison strengthened the analytical rigor by illuminating how global narratives align or fail to align with practitioner experiences on the ground.

To enhance the transparency of the analytical process, further detail on coding procedures is provided. The initial codebook was developed inductively from a close reading of five transcripts and then iteratively refined using constant comparison techniques. NVivo memos were used to document analytic decisions, theme boundaries, and reflections on potential researcher bias. Additionally, triangulation between literature-derived insights and interview-based themes was strengthened through analytic matrices that systematically compared evidence sources, ensuring that convergence and divergence were explicitly examined. The point here is that the layered analytical strategy not only increases credibility but also demonstrates coherence between data sources, thereby reinforcing the validity of the mixed-methods design.

Contradictory cases (for example, participants expressing strong enthusiasm for telemedicine despite infrastructural constraints) were systematically coded and examined as “negative cases.” Rather than being treated as anomalies, these were analyzed for the unique contextual factors that shaped them, thereby enriching thematic complexity and preventing overgeneralization. Negative case analysis played a crucial role in refining theme boundaries and ensuring analytic precision.

### Ethical considerations

All participants provided informed consent, and data were anonymized to ensure confidentiality. Participants were made aware that findings would be reported in aggregated form, maintaining their privacy while enabling insights relevant to broader digital health discussions. All procedures adhered to established ethical guidelines for qualitative research, including voluntary participation, right to withdraw, and secure data storage.

Additional safeguards were incorporated during fieldwork. Interviewer emphasized that participation or refusal would have no implications for clinical employment or professional standing. Care was taken to schedule interviews at times convenient for practitioners to minimize disruption to clinical duties. In addition, participants were provided with opportunities to clarify or amend their statements during or after the interview, supporting autonomy and ensuring that reported insights accurately reflected their intended meaning.

Finally, to uphold international standards for data stewardship, all raw and processed data were stored in compliance with principles of minimum necessary access, encryption, and secure disposal timelines. These enhanced ethical protocols reflect a commitment to responsible qualitative practice.

### Content analysis (narrative review of literature)

The literature on digital health has expanded rapidly over the past decade, with an exponential surge following the onset of the COVID-19 pandemic. This section synthesizes key strands of research on the transition toward digital healthcare, focusing on digital health literacy, the digital divide, trust and adoption dynamics, and comparative global case experiences.

1. Defining digital health literacy

Digital health literacy is widely understood as the intersection of digital literacy and health literacy; requiring individuals to access, understand, and effectively apply digital technologies in health contexts (Yang *et al*., 2022). While earlier conceptualizations of eHealth literacy focused on basic internet navigation and comprehension (Norman and Skinner, 2006), more recent frameworks incorporate advanced competencies, such as interpreting wearable device data, navigating electronic health records, and engaging with AI-driven diagnostic tools (Palumbo *et al*., 2022).

It is critical to note that inadequate digital health literacy can compromise patients' ability to understand diagnostic information, manage medications correctly, and seek timely care, all of which may increase risks to patient safety (World Health Organization, 2025; Morrison *et al*., 2021; Wamala Andersson and Gonzalez, 2025). In broad sense, the literature increasingly frames digital health literacy not only as a facilitator of technology adoption but as a prerequisite for safe and effective care delivery.

However, the depth of digital skills required varies considerably by context. In high-resource settings, digital health literacy may involve managing complex patient portals, while in low-resource environments it might mean mastering SMS-based health alerts. This variability underscores the need for context-specific definitions and measurement tools; an area that remains underdeveloped in the literature (Kickbusch, 2001; Manteghinejad and Javanmard, 2021).

- Digital health and the COVID-19 Catalyst

The pandemic acted as both an accelerant and a stress test for digital health systems. Teleconsultations, digital triage tools, and mobile vaccination certification apps became commonplace within months (Sui and Facca, 2020; Sentell *et al.*, 2020). However, the rapid rollout often compromised usability, accessibility, and data security (Polzer and Goncharenko, 2022).

It is important to highlight that rapid deployment without consideration of socio-technical readiness can threaten patient safety. Systems implemented without adequate guidance, training, or accessibility support may increase the risk of incorrect data entry, miscommunication, or inappropriate clinical decision-making, all of which can lead to adverse patient events (WHO, 2019).

The Qatar Ehteraz app, for instance, was lauded for integrating vaccination status and contact tracing in a single interface, but also highlighted inequities as those without smartphones or sufficient technical literacy were effectively excluded from accessing public spaces (Al Khal *et al.*, 2020; Hafizh *et al*., 2022). In the UK, the NHS COVID-19 app suffered from privacy concerns and technical glitches that diminished public confidence (Polzer and Goncharenko, 2022).

These examples illustrate that even technologically advanced solutions may compromise patient safety if adoption is uneven or trust is lacking. It infers that equitable, context-aware implementation is essential for ensuring that digital health interventions improve outcomes rather than introduce new risks.

In many LMICs, the pandemic spurred first-time digital health adoption among populations previously unexposed to such technologies. While this created a short-term boost in digital health literacy, studies suggest the effect was uneven and often temporary, particularly when driven by compliance rather than voluntary uptake (Zakar *et al*., 2021). Fellner *et al.* (2025) emphasize that effective digital health adoption depends on governance, infrastructure, workforce capacity, and community engagement. OECD (2025) indicates that access to electronic health records and telemedicine services has expanded across health systems post-pandemic, but interoperability and sustained use remain challenges for equitable adoption. Fellner *et al.* (2025) also emphasize that effective digital health adoption depends on governance, infrastructure, workforce capacity, and community engagement. Even in high-income countries, technical readiness and patient digital skills vary, showing that structural and socio-technical barriers are universal determinants of effective adoption. This variation is reflected in cross-national indicator evidence showing substantial differences in access to electronic health records, use of telemedicine, and self-reported confidence in using digital health tools across OECD countries (Fellner *et al.*, 2025).

- The Digital Divide as a Structural Determinant

The digital divide remains a persistent determinant of health inequity, despite global internet penetration reaching 67% in 2023 (International Telecommunication Union, 2023). The divide is not merely about access to devices or connectivity but extends to skills, confidence, and cultural fit with digital tools (Aissaoui, 2022).

Evidence indicates that even when access barriers are removed, disparities in actual use persist. In a controlled US study, participants provided with tablets and internet access demonstrated varying levels of engagement, with age, education, and prior digital experience shaping outcomes (Walker *et al*., 2020). Similarly, in Israel, eHealth literacy disparities were found to map closely onto existing socio-economic and demographic inequalities (Neter and Brainin, 2012).

Language barriers amplify these inequities. With the majority of online health information produced in English, non-English-speaking populations face structural exclusion (Kickbusch, 2001). While some digital health interventions have incorporated multilingual support, this is often partial and does not address deeper cultural and literacy considerations (Chang *et al*., 2004).

These structural inequities directly impact patient safety. Patients who cannot effectively use digital platforms may experience delays in care, errors in self-monitoring, and increased reliance on informal or unsafe health practices. These risks are particularly acute in LMIC settings, where healthcare systems may not provide redundant safety checks.

- Trust, privacy, and public perception

Trust is a critical determinant of digital health adoption (Polzer and Goncharenko, 2022). Public skepticism often revolves around data privacy, security breaches, and the potential misuse of personal health information. In the UK, lack of transparency in the development and governance of the COVID-19 app contributed to low uptake. In LMICs, mistrust can be compounded by prior experiences with government surveillance or data exploitation (Zakar *et al*., 2021).

A critical patient safety angle emerges here: when patients do not trust digital systems, they may avoid using platforms for essential health monitoring or miss important health alerts, resulting in potential adverse outcomes. OECD (2023) underscores that trust in digital systems is contingent on transparent governance, interoperability standards, and clear communication with users. OECD (2025) highlights that confidence in using digital health tools varies across demographic groups, with older and less educated individuals reporting lower confidence and greater difficulty understanding health information, which may influence uptake and trust. Health systems that fail to integrate these socio-technical factors risk underutilization and potential safety hazards, even where technology is available. Ensuring trustworthy design, transparent governance, and community engagement is therefore a safety imperative (World Health Organization, 2021).

A recent scoping review found that performance expectancy (akin to perceived usefulness), effort expectancy (akin to ease of use), and trust were among the principal determinants of engagement with telemedicine and other digital health services, underscoring that these socio-technical factors shape sustained uptake (Malaise and Mohammed, 2025). Nevertheless, trust-building is resource-intensive and requires clear communication, active community engagement, and transparent governance frameworks. UK NHS app abandoned over surveillance concerns (Polzer and Goncharenko, 2022); trust predicts engagement with digital health platforms (Dowthwaite *et al*., 2021).

- Global Case Comparisons

United States: Telehealth adoption peaked during the early pandemic but has since stabilized, with persistent disparities by age, race, and geography (Ramsetty and Adams, 2020; Wakeman *et al.*, 2025). Reimbursement policies and state-level regulatory variations continue to shape its future trajectory.

India: The Ayushman Bharat Digital Mission aims to create a unified national digital health ecosystem, including health IDs and interoperable health records. While ambitious, its success relies on bridging significant rural–urban digital literacy gaps. (Ministry of Health and Family Welfare, 2023).

Sub-Saharan Africa: Mobile health interventions, such as SMS-based maternal health reminders, have shown promise in improving care uptake, but scalability is often hampered by infrastructure limitations and sustainability concerns (Osei *et al.*, 2021).

Nordic countries: With high baseline digital literacy, countries like Finland and Denmark have implemented nationwide electronic health record systems successfully. However, even here, digital exclusion still persists among elderly and migrant populations (Mubarak and Suomi, 2022).

Each case demonstrates that inequitable adoption, limited digital literacy, and low trust can lead to gaps in safe care delivery, adverse events, and suboptimal health outcomes. These country-level experiences align with OECD (2023) analyses of digital health readiness and system-level capacity, as well as indicator-based evidence showing marked variation in digital health confidence, access to electronic health records, and use of telemedicine across population groups (Fellner *et al.*, 2025). Together, this evidence indicates that digital transformation produces equitable and safe outcomes only when infrastructure, governance, digital skills, and patient engagement are developed in parallel. Global comparisons therefore reinforce the view that digital health functions as a safety-critical socio-technical intervention rather than a standalone technological solution.

- Gaps in the Literature

Despite the proliferation of studies, several gaps remain:

1. Longitudinal evidence on whether pandemic-driven digital health literacy gains are sustained over time.
2. Intersectional analyses examining how digital exclusion intersects with gender, disability, and migration status.
3. Comparative policy evaluations of national digital health strategies.
4. Limited evaluation of co-design models in which end-users actively shape digital health tools.

It should be emphasized here that gaps in digital literacy, equity, and trust are not abstract; they have direct implications for patient safety. Future research must link adoption outcomes with measures of care quality, adverse events, and equitable access to ensure digital health fulfills its promise as a safe and effective modality.

Overall, this review highlights that transitioning to digital health is not merely a technological deployment; it is a complex socio-technical process requiring careful alignment among innovation, equity, and trust. Critically, this alignment is directly linked to patient safety: technology adoption without equity and literacy risks compromising care outcomes.

## Results

### Integration of findings

Results from the literature review and interviews were integrated at the interpretation stage using a side-by-side comparison approach (Fetters *et al.*, 2013). This method allowed for the identification of convergences (e.g., universal patient digital skills gaps) and divergences (e.g., trust and privacy concerns being more prominent in high-income contexts). Explicitly comparing local practitioner experiences with global literature, author was able to highlight context-specific nuances and identify where Karachi reflects broader LMIC patterns versus unique local challenges.

Consistent with OECD (2023) analysis, health systems’ readiness for digital transformation depends on robust infrastructure, governance, and workforce capacity, highlighting structural barriers that can limit equitable adoption even when technology is available. Importantly, these findings were also examined for implications on patient safety, identifying where gaps in digital literacy, infrastructure, or system usability could translate into adverse care events.

### Results overview

The combined analysis of the literature review and the 31 practitioner interviews reveals a complex landscape in which digital health adoption is both enabled and constrained by interrelated socio-technical factors. The results are presented under five primary themes. Each theme is linked explicitly to the research questions, emphasizing how structural, cultural, and technical factors shape adoption. Where relevant, connections to patient safety outcomes are highlighted.

### Theme 1: Technical Barriers and Application Failures

A recurring point from both the literature and interviews was the prevalence of technical malfunctions in digital health tools, particularly during the pandemic's initial response phase.

1. Interviewees described frequent application crashes, login failures, and server downtime during periods of high demand.
2. Some mobile applications (introduced to facilitate teleconsultations or vaccination record-keeping) were reported as “barely functional” in low-bandwidth areas.
  - Differences were observed between hospital types: practitioners in private hospitals reported slightly better connectivity and app stability than those in public facilities, highlighting infrastructural disparities.

These issues are echoed in global cases:

1. The UK NHS COVID-19 app required repeated updates to address software bugs, undermining user trust (Polzer and Goncharenko, 2022).
2. In LMIC contexts, intermittent electricity supply and limited 4G coverage exacerbated service downtime (Ahmed *et al*., 2023). OECD (2023) data indicate that even in high-income countries, intermittent connectivity, server downtime, and fragmented digital infrastructure have hindered consistent digital health use, showing that technical reliability is a universal prerequisite for adoption.

*Interpretation*: Technical reliability is a prerequisite for adoption; functionality failures reduce engagement and erode trust, particularly in contexts with limited infrastructural resilience. Importantly, these failures may compromise patient safety by delaying access to care, causing miscommunication, or preventing timely clinical decisions, linking directly to RQ1.

### Theme 2: Patient Digital Skills Gap

The most consistently cited barrier mentioned by 98% of interviewees was the patient skills gap.

1. Even patients who could operate smartphones struggled with tasks like uploading documents, creating accounts, or troubleshooting login errors.
2. This challenge was most acute among older adults, individuals with lower formal education, and first-time smartphone users.
  - Practitioners highlighted that family support often mediates adoption, but reliance on intermediaries can introduce delays or errors, demonstrating an additional layer of inequality.

Global evidence aligns with these observations:

1. In the US, older adults remain less likely to engage with patient portals, even when provided access and training (Walker *et al*., 2020).
2. In rural Turkey *et al.* (2022) found that mobile health app users demonstrated significantly higher health literacy than non-users, underscoring the self-reinforcing nature of skills gaps. OECD (2023) findings further emphasize that disparities in digital literacy are a persistent barrier, with gaps in patient skills and engagement affecting equitable adoption across countries.

“They can answer a call or send a message, but when you ask them to upload a vaccination certificate, they give up.” – Male GP, age 45 > “Older patients often rely entirely on family members to navigate apps, which slows adoption and increases inequality.” – Female specialist, age 38.

*Interpretation:* Digital literacy is a central determinant of adoption. Addressing skill gaps requires both patient education and interface simplification. Insufficient skills may not only impede adoption but also compromise safe care delivery, e.g., incorrect reporting of symptoms, miscommunication in teleconsultations, or delayed access to critical health information, thereby linking this theme to RQ2 and patient safety.

Theme 3: Cultural and Language Barriers

Cultural misalignment and language exclusivity emerged as substantial obstacles:

1. Many COVID-era applications in Pakistan and other LMICs were available only in English, excluding non-English speakers or those with limited literacy.
2. Even when translated, technical jargon often limited usability.
  - Participants noted that even simple wording changes in Urdu or Sindhi significantly improved comprehension, highlighting the importance of iterative, user-informed localization.

### Literature support

1. Kickbusch (2001) highlighted the linguistic dominance of English in health information as a structural exclusion factor.
2. In Qatar, the Ehteraz app interface was available in Arabic and English, yet migrant workers with neither language faced exclusion (Hafizh *et al*., 2022).

*Interpretation:* Cultural and linguistic tailoring is essential to ensure equitable access. Failure to localize content can compromise patient safety by creating misinterpretation of health instructions, improper use of digital tools, or errors in symptom reporting. This links to RQ2 and RQ3, emphasizing that culturally aligned systems are necessary for both adoption and safe care.

### Theme 4: Trust and Data Privacy Concerns

Concerns about data privacy were more prominent in the literature than in the Pakistani interview context, where urgency of access often outweighed privacy considerations:

1. UK users abandoned the NHS app over fears of surveillance (Polzer and Goncharenko, 2022).
2. In contrast, several Karachi practitioners noted that patients “rarely asked about data safety,” focusing primarily on functional reliability.
  - Some practitioners expressed caution in assuming privacy concerns were absent, noting that awareness may grow as digital literacy increases.

*Interpretation:* Trust concerns are context-dependent. While globally privacy can inhibit adoption, in low-resource settings practical utility and reliability dominate. Nonetheless, breaches of privacy or inadequate security could indirectly affect patient safety if sensitive data is lost or misused. This theme addresses RQ2 by showing how trust interacts with socio-technical readiness and care quality.

Theme 5: Policy and Systemic Alignment

Practitioners emphasized that digital health would only succeed if integrated into a broader digital ecosystem, including:

1. Digital literacy training programs
2. Affordable and reliable internet access
3. Government-backed technical support channels

Literature reinforces these needs:

1. World Health Organization (2025) policy briefs and evidence from empirical research (Wilson *et al*., 2024) stress co-design with end-users, especially marginalized groups. Fellner *et al.* (2025) similarly highlight that digital health adoption requires coherent policy frameworks, interoperability standards, and institutional support to ensure tools are effective and accessible, reinforcing the importance of system-level alignment for equitable adoption.
2. Palumbo *et al*. (2022) argue that one-size-fits-all approaches fail without customization to community needs.
  - Interviewees highlighted that without institutional support and ongoing maintenance, even well-designed apps fail to scale.

*Interpretation:* Policy frameworks and system-level support are critical enablers. Absence of alignment can indirectly compromise patient safety by preventing consistent monitoring, creating gaps in follow-up care, or limiting access to reliable health information, linking this theme to RQ3.

### Integrated interpretation in a nutshell

When integrating the literature and interview findings, three cross-cutting insights emerge:

(1) Digital health is more likely to fail in the absence of foundational digital inclusion policies including infrastructure, skills, and trust-building.
  - Practitioners noted that short-term fixes, like temporary subsidies or emergency training, are insufficient for long-term adoption. *Such foundational weaknesses also pose potential risks to patient safety if errors, delays, or miscommunications occur due to technical or skills-related barriers.*
(2) Mandated adoption during the pandemic created short-term familiarity but did not necessarily produce sustained literacy gains.
  - Continued exposure and reinforcement are necessary to convert familiarity into competent usage.
(3) Socio-cultural tailoring including language adaptation and interface simplification—is crucial for equitable uptake.
  - Iterative feedback loops with local users enhance engagement and ensure tools meet diverse needs. *Neglecting these adaptations could increase patient safety risks, especially among vulnerable or low-literacy populations.*

### Integrated interpretation

The integration of literature and interview findings demonstrates that the success of digital health adoption is fundamentally dependent on robust digital inclusion policies. Infrastructure, digital skills, and trust-building emerge as interdependent foundations for equitable uptake, rather than optional supports. Practitioners consistently emphasized that short-term interventions (such as emergency training sessions or temporary subsidies) are insufficient to produce lasting change. Without sustained investment in these foundational elements, even technically sound digital tools risk underutilization or abandonment, particularly in resource-constrained LMIC contexts like Karachi. Critically, deficits in these foundations can directly affect patient safety, potentially resulting in delayed care, miscommunication, or misuse of digital tools.

Pandemic-driven mandates increased immediate familiarity with digital health applications; however, this familiarity did not automatically translate into competence, confidence, or long-term adoption. Both the literature and practitioner accounts suggest that repeated exposure, ongoing support, and iterative learning opportunities are essential to convert short-term engagement into sustained digital literacy. Digital health adoption is therefore a processual endeavor, requiring reinforcement at multiple levels of the healthcare ecosystem, from individual users to institutional frameworks. Proper localization and user-informed design are not only adoption facilitators but also safeguards for patient safety, preventing misinterpretation or incorrect use of digital health tools.

Equally critical is the socio-cultural tailoring of digital health tools. Language adaptation, interface simplification, and alignment with local norms were repeatedly cited as decisive factors in patient engagement. Iterative feedback loops with local users and culturally responsive modifications significantly enhance adoption, particularly among populations with lower literacy or limited prior exposure to technology. In Karachi, for instance, practitioners noted that even minor localization in Urdu or Sindhi improved comprehension and usability, illustrating the importance of context-sensitive design.

Together, these findings illustrate that digital health cannot fulfill its promise without a holistic, integrated approach; one that simultaneously addresses technical functionality, human competencies, and systemic support. Explicitly linking global patterns from the literature with the lived experiences of practitioners in Karachi, this study underscores that sustainable adoption depends on aligning digital innovation with local realities, thereby bridging the gap between technological potential and real-world implementation. These insights are consistent with OECD (2023) and Fellner *et al.* (2025), which indicate that successful digital transformation depends not only on technology deployment but also on governance capacity, workforce readiness, and equitable access, underscoring that socio-technical preparedness is critical for meaningful adoption and patient safety. In this context, gaps in governance, skills, or access directly threaten equitable uptake and may compromise patient safety, highlighting why socio-technical readiness must underpin all digital health initiatives. Table 1 brings together the main themes, the supporting interview evidence, the corresponding literature, and their relevance to the research questions.

**Table 1:** Mapping findings with literature

Theme	Interview evidence	Literature evidence	Interpretation / link to RQs
1. Technical barriers and application failures	Practitioners reported frequent app crashes, login failures, and server downtime. Differences were observed between hospital types, with private hospitals reporting better connectivity and application stability than public facilities	UK NHS COVID-19 app required repeated updates; intermittent electricity and limited 4G exacerbated service downtime in LMICs (Polzer and Goncharenko, 2022; Ahmed *et al*., 2023; OECD, 2023)	Technical reliability is essential for adoption. Functionality failures reduce engagement and trust. RQ1: Shows how infrastructure and technical failures inhibit equitable uptake
2. Patient digital skills gap	Patients struggled with uploading documents, creating accounts, troubleshooting; older adults and low-education groups most affected; reliance on family intermediaries	Older adults in US remain less likely to engage with portals (Walker *et al*., 2020), rural Turkey users had higher eHealth literacy than non-users (Sariyildiz *et al*., 2022; OECD 2023) highlights that digital health adoption depends on general digital readiness, including user skills and capacity; building workforce capacity is crucial for meaningful digital health adoption Fellner *et al.* (2025)	Digital literacy is central for adoption. Addressing skills gaps requires patient education and simplified interfaces. RQ2: Highlights socio-technical mediation of uptake
3. Cultural and language barriers	Many COVID-era applications were available only in English. Practitioners reported that wording changes in Urdu or Sindhi improved patient comprehension	English dominance in health information excludes non-speakers (Kickbusch, 2001); Qatar Ehteraz app excluded some migrant workers (Hafizh *et al*., 2022)	Localization and cultural tailoring are crucial. RQ2 & RQ3: Impacts adoption and sustainability through cultural fit
4. Trust and data privacy concerns	Patients rarely asked about data safety; functional reliability prioritized	UK NHS app abandoned over surveillance concerns (Polzer and Goncharenko, 2022), trust predicts engagement with digital health platforms (Dowthwaite *et al*., 2021; OECD 2023)	Trust concerns are context-dependent; in low-resource settings, practical utility dominates. RQ2: Shows interaction of trust and socio-technical readiness
5. Policy and systemic alignment	Need for digital literacy programs, affordable internet, government-backed support; apps fail without institutional backing	WHO (2025) and Wilson *et al*. (2024) emphasize co-design; Palumbo *et al*. (2022) stress community customization; Fellner *et al.* (2025) highlight governance, infrastructure, skills, and engagement	System-level support and policy frameworks enable sustainable integration. RQ3: Highlights strategies for long-term adoption

## Discussion

The global transition to digital health has often been portrayed as both inevitable and transformative, a trajectory accelerated by the unprecedented disruptions of the COVID-19 pandemic. In practice, however, the evidence from this study drawing on both scholarly literature and firsthand accounts from healthcare practitioners in Karachi; reveals that this transformation is uneven, context-dependent, and mediated by structural, cultural, and technical factors. The findings highlight a recurring disjuncture between technological optimism and practical outcomes, particularly in settings marked by socio-economic and infrastructural inequalities. Importantly, these gaps have direct implications for patient safety, as unreliable systems, low digital literacy, and cultural misalignment can compromise the quality and safety of care delivered. By situating these insights within broader scholarly debates, this discussion underscores the socio-technical complexity of digital health adoption and clarifies implications for policy, practice, and future research.

A key theme emerging from the integrated results is that digital health cannot be understood solely as a technological innovation; it must instead be conceptualized as a socio-technical process shaped by both material infrastructures and social determinants. While technologies promise improved efficiency and accessibility, their effectiveness is mediated by contextual factors such as literacy, income, geography, and cultural norms. The five main themes identified in the results (technical barriers, patient digital skills gaps, cultural and language barriers, trust and privacy concerns, and policy/systemic alignment) provide a structured lens to understand these complexities. Each of these factors also intersects with patient safety: technical failures can lead to delays or errors in treatment, digital literacy gaps may cause incorrect patient self-management, and cultural misalignment can reduce adherence to care protocols.

The interviews conducted in Karachi, combined with global case evidence, underscore the fragility of many digital health initiatives when usability, accessibility, and reliability are not adequately addressed. The difficulties encountered during the rollout of the NHS COVID-19 app, plagued by software bugs and usability concerns, exemplify how technical fragility can erode trust and undermine uptake (Polzer and Goncharenko, 2022). Such technical issues, if unaddressed, can result in miscommunication, missed diagnoses, or delayed interventions, thereby directly affecting patient safety. In lower-resource settings, where device incompatibility and intermittent internet coverage are common, these technical issues compound existing inequities, disproportionately affecting marginalized populations (Ahmed *et al*., 2023).

Closely linked to technical barriers is the pervasive patient digital skills gap, which emerged as the most frequently cited obstacle in both interviews and literature. The evidence demonstrates that provision of hardware and connectivity alone is insufficient; users must also develop operational, navigational, and evaluative digital health skills. Many patients struggle with basic functions, such as uploading vaccination certificates or creating accounts, while more advanced tasks (like interpreting health data or evaluating the reliability of information) remain challenging. This layered skills gap underscores the need for sequenced and sustained digital health literacy interventions, moving from foundational training to higher-level competencies. Addressing these gaps is critical for patient safety, as incorrect use of digital health tools can lead to misreporting of symptoms, medication errors, or improper follow-up. Without such structured approaches, well-intentioned programs risk entrenching rather than alleviating exclusion (Walker *et al*., 2020; Sariyildiz *et al*., 2022).

Cultural and linguistic factors constitute another critical determinant of effective adoption. While translation of digital tools is often assumed to suffice for inclusivity, the study highlights that mere linguistic adaptation does not guarantee usability or engagement. Persisting technical jargon, bureaucratic terminology, and culturally unfamiliar interfaces can exclude large segments of the target population (Kickbusch, 2001; Hafizh *et al*., 2022). The cases of Qatar's Ehteraz app and India's Ayushman Bharat Digital Mission illustrate that even when language options are provided, effective adoption requires contextualized, community-based training and culturally sensitive implementation strategies. *Failure to address these factors can compromise patient safety, for example when patients misinterpret instructions or fail to follow prescribed care pathways.* This finding emphasizes that digital health equity depends on holistic design approaches that integrate cultural relevance into technological development.

Trust and data privacy concerns emerged as contextually dependent factors. In high-income countries, privacy apprehensions and fears of surveillance can significantly hinder uptake, as evidenced by users abandoning the NHS app (Polzer and Goncharenko, 2022). In the NHS COVID-19 app study, users who reported lower trust in the system were also less likely to adopt the app, indicating that trust shapes uptake and user attitudes toward digital health tools (Dowthwaite *et al*., 2021). In contrast, in the Karachi context, patients prioritized functional access over privacy concerns; interviewees noted that patients rarely questioned data safety and instead focused on whether applications were operational. This divergence highlights the importance of context-sensitive and anticipatory governance frameworks, suggesting that trust-building strategies must be tailored to local priorities while remaining forward-looking to anticipate future data sensitivity as digital ecosystems evolve. From a patient safety perspective, transparent communication about data handling and secure systems ensures that health information is accurate, reliable, and used appropriately, preventing harm from misinformation or breaches.

The pandemic further demonstrated how mandates can temporarily accelerate digital health adoption. While enforced usage such as through travel requirements or vaccination verification expanded initial exposure to digital tools, these gains were often compliance-driven and transient. Both the literature and interviews indicate that without sustained engagement, simplified user support, and community-based reinforcement, such mandated adoption does not translate into lasting digital literacy or behavior change. To convert short-term exposure into durable competencies, health systems must complement mandates with ongoing education, technical support, and iterative co-design with end-users. Sustained engagement is essential to patient safety, as intermittent or superficial adoption may leave patients ill-equipped to use digital tools safely and effectively.

Another insight relates to the necessity of systemic alignment for successful digital health implementation. Practitioners emphasized that isolated technological rollouts are insufficient in the absence of a broader ecosystem of support, including infrastructure, digital literacy programs, and policy alignment. This finding, reinforced by WHO (2025), Wilson *et al.* (2024), Palumbo *et al.* (2022), and Fellner *et al.* (2025), underscores that digital health adoption requires integration into national digital transformation agendas and cross-sectoral investment in skills, connectivity, governance, and stakeholder engagement. Without such sustained and coordinated alignment, digital health initiatives risk being underfunded, fragmented, or accessible primarily to advantaged populations. As well, without these systemic supports, digital health interventions risk introducing errors or inequities that compromise safe care delivery.

Ultimately, this study reinforces the principle that innovation without equity is insufficient. Digital health interventions that fail to consider socio-technical prerequisites may inadvertently reproduce existing disparities. Conversely, when tools are co-designed with end-users, culturally and linguistically adapted, and embedded within supportive policy and infrastructural frameworks, digital health can enhance inclusion and equity. Co-production approaches, though resource-intensive, are more likely to produce trusted, effective, and contextually relevant solutions that achieve both practical utility and sustainability.

**Figure 2:**
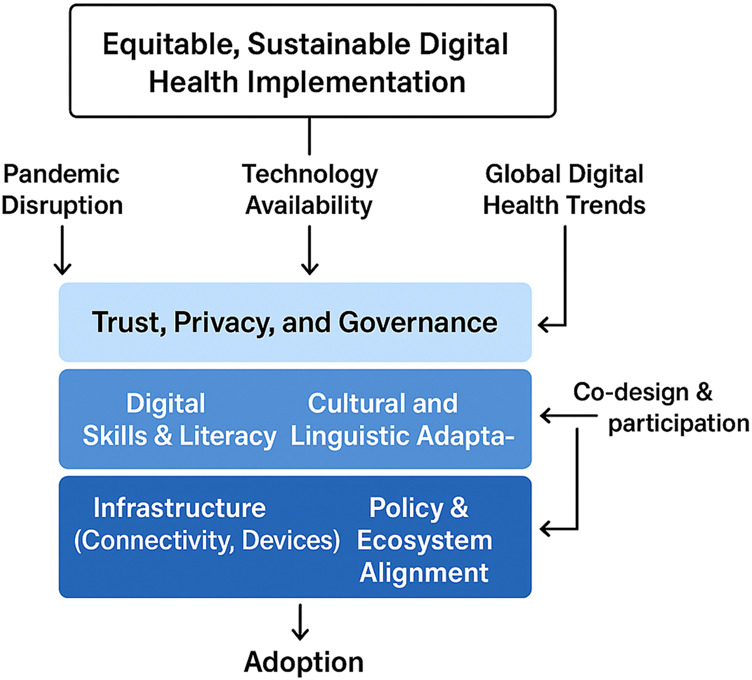
Socio-technical framework for equitable digital health adoption

Taken together, these findings emphasize that digital health must be approached as a systemic transformation rather than a purely technical innovation. This systemic perspective is synthesized in Figure 2, which presents a socio-technical framework for equitable digital health adoption, illustrating how infrastructure, digital literacy, trust, and participatory design processes interact to shape inclusive implementation outcomes. Realizing its potential requires careful attention to OECD-recommended governance, workforce, infrastructure, and engagement measures (OECD, 2023, 2025). Equity at the center of digital health implementation enables policymakers and practitioners to move from the rhetoric of inevitability toward the reality of inclusive, effective, safe, and sustainable healthcare innovation.

Figure 2 presents the key factors that shape the adoption of digital health in a way that is inclusive and safe. Four core elements are central to the framework: infrastructure, digital literacy and skills, trust and data privacy, and participatory design with users. Each of these elements interacts with the others. For example, reliable infrastructure enhances trust, while participatory design supports the development of digital skills. These interactions are represented with connecting arrows to illustrate the continuous feedback between elements.

Patient safety and quality of care are shown as the primary outcomes of this system. All four socio-technical elements influence these outcomes, demonstrating that adoption is not solely determined by technology but by the combination of social, technical, and policy factors. Beneath these elements lies a foundational layer of policy, governance, and workforce capacity, reflecting the recommendations of the OECD and the World Health Organization. This layer supports the effective functioning of the framework by ensuring that infrastructure, skills development, and trust-building are sustained.

Equity and inclusion are central to the framework. Every component is designed to support populations with varying levels of literacy, digital experience, and socio-economic status. The framework emphasizes that equitable digital health adoption requires attention to both the design of tools and the systems in which they operate.

## Conclusion and implications

The COVID-19 pandemic marked a historic inflection point for digital health, transforming it from a gradual reform trajectory into an urgent and central strategy for healthcare delivery. The global health crisis forced healthcare systems, regardless of resource level, to experiment with new ways of delivering care, ranging from teleconsultations and mobile vaccination records to AI-assisted diagnostics and wearable health-monitoring devices. This unprecedented acceleration demonstrated not only the adaptability of health systems under pressure but also the fragility of existing structures when confronted with rapid technological change. The pandemic, in effect, functioned as a large-scale stress test, revealing both the potential and the vulnerabilities inherent in digital health ecosystems. It also highlighted critical implications for patient safety, as hurried or poorly integrated digital tools risked miscommunication, diagnostic delays, and compromised care quality.

The premise driving this shift was that digital platforms could serve as equalizers, reducing physical, temporal, and financial barriers to healthcare access. However, the findings from this study reveal a stark divergence between technological potential and real-world outcomes. Both the literature review and the interview data show that without addressing foundational socio-technical prerequisites, digital health adoption risks reproducing (and in some cases exacerbating) existing health disparities. In other words, digital health is not inherently democratizing; its success depends on the inclusiveness, integrity, and readiness of the ecosystems into which it is introduced. Moreover, safe care practices are contingent on ensuring that digital adoption does not compromise clinical decision-making, data accuracy, or continuity of care.

### Restating the core findings

Persistent Digital Divide – Despite global gains in connectivity, the divide has not closed; it has evolved. It now encompasses not only access gaps but also skills, trust, and cultural fit, all of which influence whether digital health tools are used effectively. This evolving divide underscores that digital exclusion is no longer simply about device ownership but increasingly about navigating complex digital environments with confidence and competence. From a patient safety perspective, unequal access can translate into uneven monitoring, delayed interventions, and differential outcomes.

Digital Skills Gap as the Critical Bottleneck: The evidence overwhelmingly points to digital skills, rather than hardware or internet access alone, as the most critical determinant of whether digital health solutions succeed or fail. Users' ability to interpret health information, operate digital interfaces, and evaluate the credibility of sources emerged as central to both meaningful engagement and sustained utilization. Without deliberate efforts to build these capacities, digital health tools risk remaining underutilized or misused. Insufficient digital literacy can also jeopardize safe care, as errors in data entry, misunderstanding of clinical advice, or misinterpretation of alerts can lead to adverse patient outcomes.

Cultural and Linguistic Relevance: Digital health interventions that fail to accommodate the linguistic and cultural realities of their intended users risk excluding large population segments. The data show that even well-designed tools can be rendered ineffective if they do not reflect the norms, communication styles, and expectations of their users. Culturally inappropriate design not only reduces engagement but may also erode trust, highlighting the need for localized, participatory approaches.

Trust and Data Governance: In high-income contexts, privacy and data security concerns can be deal-breakers for adoption. In low-income contexts, they may be temporarily overlooked, but they will become increasingly important as systems mature. Trust, therefore, operates as a cross-cutting determinant that shapes not only initial uptake but also long-term adherence to digital health practices. Building trust early through transparency, clear communication, and robust governance is essential for sustainable digital engagement. Trust also underpins patient safety, as secure and reliable systems prevent errors and support accurate, timely care delivery.

Policy and Ecosystem Readiness: Technology rollouts are most successful when embedded in a broader policy ecosystem that includes education, infrastructure, and sustained user engagement. Fragmented or top-down implementations consistently struggle to reach vulnerable populations, reinforcing the need for cohesive governance structures. Integration across sectors (health, education, and technology) is therefore essential to maximize the impact and sustainability of digital health initiatives. Sustained benefits require that these interventions are embedded within ongoing education, infrastructure development, and participatory governance, rather than implemented as temporary or isolated projects.

### Implications

The integration of digital health into national development agendas presents both an opportunity and a necessity, particularly for low- and middle-income countries where health inequities remain pronounced. Positioning digital health within broader digital transformation strategies ensures that investments in connectivity, education, and digital skills deliver tangible public health benefits rather than remaining isolated technological projects. Such cross-sectoral alignment prevents fragmentation and enhances the sustainability of digital health interventions. Moreover, aligning digital health with national policy priorities creates the institutional stability required for long-term reform.

However, the success of these strategies depends on the adoption of participatory design approaches that actively involve end-users (particularly marginalized and vulnerable groups) in the development and testing of digital health tools. Co-design not only enhances usability and cultural resonance but also builds trust and fosters a sense of ownership, which are essential for widespread adoption. Participants consistently emphasized that when users are treated as active partners rather than passive recipients, their willingness to adopt digital health tools increases significantly. Importantly, participatory design also mitigates patient safety risks by ensuring that tools are intuitive, error-resistant, and aligned with clinical workflows.

Equally critical is the elevation of digital literacy as a public health priority. Digital skills training for patients, clinicians, and community leaders should be institutionalized across formal education systems and reinforced through informal community-based outreach. Embedding capacity-building efforts at multiple levels ensures that both providers and users are empowered to engage effectively with digital tools. Context-specific, language-appropriate, and incremental training is essential for narrowing the skills gap identified in this study. Effective training directly contributes to safe care by reducing errors and enhancing proper utilization of digital health interventions.

Alongside these efforts, robust data governance frameworks are indispensable. Transparent, enforceable policies for data protection and privacy safeguard public trust, drawing on international standards while being adapted to local legal and cultural contexts. Without such regulatory scaffolding, digital health risks exacerbating mistrust and reinforcing existing inequities. Clear communication about how data are collected, stored, and used can mitigate fears that often deter individuals from engaging with digital systems. Proper governance also underpins patient safety by ensuring that health data remain accurate, accessible, and secure. Supporting people to use digital health tools, designing them to fit local culture, and having clear and reliable governance all help ensure these tools are adopted widely and used safely.

The translation of these policy commitments into practice requires parallel investments in the healthcare workforce and service delivery infrastructures. Healthcare professionals must not only be proficient in digital tools but also act as mediators, guiding patients through adoption and use. Their attitudes, confidence, and communication strategies are pivotal in translating technology into practical healthcare benefits. Workforce competence is a key determinant of both safe care and equitable digital health outcomes.

Localized content development is equally essential, ensuring that health applications are available in multiple languages and adapted to the literacy levels of target populations. Collaborative work among health educators, linguists, and community representatives can ensure accessibility and cultural appropriateness. Complementing these measures, practical support infrastructures (such as toll-free helplines, digital navigators, and on-site assistance points) act as transitional mechanisms that reduce frustration, prevent dropout, and bridge the digital divide. Such mechanisms also prevent missteps and enhance safe, correct use of digital tools, thereby protecting patients.

These developments also create new research imperatives. Longitudinal studies are needed to examine whether digital health gains achieved during the pandemic can be sustained over time and under what conditions. Intersectional approaches are essential to understand how digital exclusion interacts with gender, disability, migration status, and chronic illness. Comparative policy research can further illuminate how diverse regulatory, financing, and implementation models influence equity and sustainability across national contexts. Such evidence is critical for guiding investments and avoiding repeated pitfalls observed during the pandemic.

**Figure 3:**
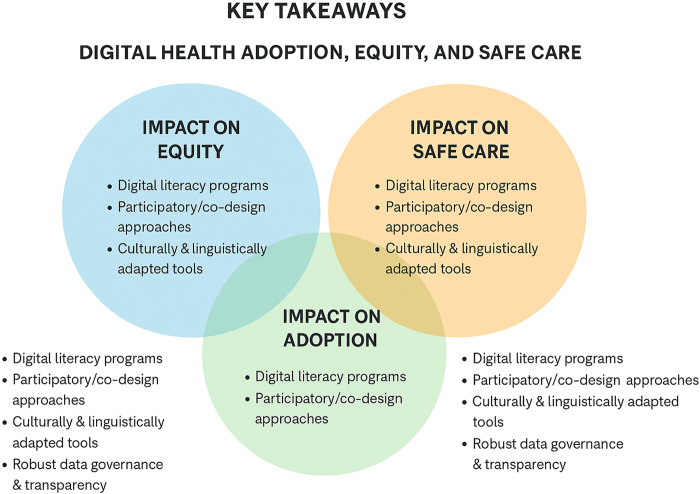
Healthcare adoption schematic

Taken together, these implications underscore that digital health is not merely a technological innovation but a systemic transformation that must be guided by coordinated policy frameworks, inclusive practices, and rigorous context-sensitive research. As illustrated in Figure 2, the interdependencies among infrastructure, digital literacy, trust, and participatory design are central to achieving equitable and safe digital health adoption. The evidence reinforces international recommendations that digital health adoption requires coordinated investments in governance, workforce capacity, and infrastructure, as emphasized by the OECD and WHO. Only through this integrated approach can digital health fulfill its promise of advancing equity, trust, resilience, *and safe care* in health systems worldwide. The study points toward a model of digital health that is developmental, participatory, and justice-oriented. Tables 2 and 3 tabulates the core points whereas Figure 3 inspires a schematic digital health adoption.

**Table 2:** Core finding, implication, and actionable recommendation

Core finding	Implication	Actionable recommendation
Persistent digital divide (access, skills, trust, cultural fit)	Digital exclusion is no longer just about devices; navigating digital environments is critical	Implement multi-level interventions: device access + skills training + culturally relevant platforms
Digital skills gap as critical bottleneck	Lack of skills impedes meaningful adoption and sustained use	Institutionalize digital literacy programs for patients, clinicians, and community leaders: *build workforce competence,* provide incremental, context-specific, and language-appropriate training
Cultural and linguistic misalignment	Tools that ignore cultural and linguistic context risk exclusion	Localize digital health applications; engage community representatives in co-design; simplify interfaces and avoid technical jargon
Trust and data governance	Functional access may outweigh privacy in LMICs now, but long-term trust is essential	Develop transparent data governance policies; communicate clearly on data use; ensure culturally sensitive privacy safeguards
Policy and ecosystem readiness	Fragmented digital health strategies fail to reach vulnerable populations	Integrate digital health into national digital transformation strategies; ensure cross-sectoral alignment (health, education, ICT)
Mandate-driven adoption is temporary	Short-term exposure without engagement does not produce sustained literacy	Combine mandates with sustained engagement strategies: follow-up training, digital navigators, support helplines
Participatory design enhances adoption	Engaging end-users improves usability, trust, and equity	Co-produce tools with end-users, particularly marginalized groups, to improve adoption and equity
Infrastructure and support systems	Technology rollout risks failure without complementary support	Invest in reliable connectivity, localized content, on-site assistance, toll-free helplines, and workforce training

**Table 3:** Key takeaways: digital health adoption, equity, and safe care

Implication/Intervention	Impact on equity	Impact on adoption	Impact on safe care
Digital literacy programs	Reduces exclusion by enabling marginalized users to navigate tools	Increases sustained engagement with digital health	Enhances correct use of tools, reduces errors, improves patient outcomes
Participatory/co-design approaches	Ensures tools reflect needs of diverse populations	Improves usability and trust, leading to higher uptake	Minimizes misuse, supports safety-aligned design, prevents miscommunication
Culturally and linguistically adapted tools	Expands access for non-dominant language speakers	Increases engagement and comprehension	Reduces risk of errors caused by misunderstanding or interface misalignment
Robust data governance and transparency	Protects vulnerable populations from privacy violations	Builds trust, facilitating sustained adoption	Ensures secure, accurate, and reliable health data for safe care
Infrastructure and system-level alignment	Ensures consistent access across socio-economic strata	Supports reliable and uninterrupted service use	Provides stable platform for safe delivery of digital interventions
Workforce capacity and digital competency	Supports equitable guidance for patients with lower digital skills	Enhances provider confidence and technology uptake	Clinicians can mediate safe use, preventing clinical errors
Practical support mechanisms (helplines, navigators)	Assists those with limited digital experience	Reduces dropout, improves adoption continuity	Provides real-time error prevention, supports safe navigation of tools
Ongoing monitoring and evaluation	Identifies equity gaps in real time	Informs iterative improvement, sustaining adoption	Detects safety risks early, enabling corrective actions before harm occurs
Integration into national digital health policies	Embeds equity principles in systemic planning	Facilitates coordinated and sustained adoption	Ensures governance and regulatory oversight for safe care delivery

### Looking forward

The trajectory toward digital health is irreversible. As technologies become more sophisticated (integrating predictive analytics, personalized treatment plans, and real-time monitoring), healthcare will continue to shift toward hybrid and fully digital service models. Yet, this transformation will only be meaningful if equity is treated as a foundational design principle rather than an optional add-on.

In high-income settings, this means closing the last-mile gaps for elderly, rural, and migrant populations. In LMICs, it requires building foundational infrastructure, trust frameworks, and skills pipelines to enable sustainable digital health adoption. The divergent starting points of these contexts necessitate context-specific, tailored strategies that reflect local realities.

The COVID-19 pandemic was an unprecedented stress test for healthcare systems. It revealed both the potential of digital solutions and the risks of rushing adoption without adequate preparation. The next decade offers a crucial window to learn from these lessons and invest in the slow, deliberate work of building inclusive digital health ecosystems. Failing to act risks entrenching a new form of digital health stratification.

If digital health is to deliver on its promises, its architects (whether policymakers, technologists, or clinicians) must keep equity at the core of design, implementation, and evaluation. Anything less risks turning the promise of digital health into yet another chapter in the long history of unequal healthcare access. The path forward therefore requires patience, strategic investment, and an unwavering commitment to inclusion. Digital health will risk reinforcing the very inequalities it seeks to address if equity is not central to its design and implementation. Digital health will risk reinforcing the very inequalities it seeks to address if equity is not central to its design and implementation; equity is not optional, and without it, digital health risks becoming a mirror of the very inequalities it aims to overcome.
